# Supplementary material for: JAK-Inhibitor and Type I Interferon Ability to Produce Favorable Clinical Outcomes in COVID-19 Patients: A Systematic Review and Meta-Analysis
Source: Res Sq. 2020 Aug 26:rs.3.rs-64782. Preprint. [Version 1] doi: 10.21203/rs.3.rs-64782/v1 (PMC7457585; doi:10.21203/rs.3.rs-64782/v1)
Supplement: Supplement — ary Figure 1. Funnel plots of JAK-inhibitor treatment for (A) Mortality, (B) ICU Admission, (C) Requirement of Mechanical Ventilation, (D) ARDS, and (E) Discharge Supplementary Figure 2. Funnel plots of Type I interferon treatment for (A) Mortality, (B) ICU Admission, (C) Requirement of Mechanical Ventilation, (D) Severe or Critical Disease, and (E) Discharge 95% CI lines were not presented when the random effects model was used. Supplementary Table 1. Total outcome data stratified by included study Studies presented in alphabetical order by treatment group. Supplementary Table 2. Definition of a severe or critical case in included studies for which that measure was analyzed. Studies presented in alphabetical order. Supplementary Table 3. Risk of Bias (RoB) 2 check list for detection of bias in randomized trials. Avenues of bias considered: Risk of bias arising from the randomization process, Risk of bias due to deviations from the intended interventions (effect of assignment to intervention), Risk of bias due to deviations from the intended interventions (effect of adhering to intervention), Risk of bias due to missing outcome data, Risk of bias due to measurement of the outcome, Risk of bias in selection of the reported result. (N=No; PN=Probably No; Y=Yes; PY= Probably Yes; NI= Not Indicated; NA= Not Applicable) Supplementary Table 4. Newcastle-Ottowa Scale (NOS) tool for risk of bias detection in non-randomized trials. Study characteristics examined: (1) representativeness of exposed cohort, (2) selection of nonexposed cohort, (3) exposure assessment, (4) outcome of interest not present at the start of the study, (5) comparability of cohorts, (6) outcome assessment, (7) adequacy of length of time before follow-up, and (8) adequacy of follow-up of cohorts [file SupplementalMaterialJAKiIFNCOVID.pdf]

Supplementary Figure 1. Funnel plots of JAK-inhibitor treatment for (A) Mortality, (B) ICU Admission, (C) Requirement of Mechanical Ventilation, (D) ARDS, and (E) Discharge

Supplementary Figure 2. Funnel plots of Type I interferon treatment for (A) Mortality, (B) ICU Admission, (C) Requirement of Mechanical Ventilation, (D) Severe or Critical Disease, and (E) Discharge

95% CI lines were not presented when the random effects model was used.

Supplementary Table 1. Total outcome data stratified by included study

Studies presented in alphabetical order by treatment group.

Supplementary Table 2. Definition of a severe or critical case in included studies for which that measure was analyzed.

Studies presented in alphabetical order.

Supplementary Table 3. Risk of Bias (RoB) 2 check list for detection of bias in randomized trials.

Avenues of bias considered: Risk of bias arising from the randomization process, Risk of bias due to deviations from the intended interventions (effect of assignment to intervention), Risk of bias due to deviations from the intended interventions (effect of adhering to intervention), Risk of bias due to missing outcome data, Risk of bias due to measurement of the outcome, Risk of bias in selection of the reported result. (N=No; PN=Probably No; Y=Yes; PY= Probably Yes; NI= Not Indicated; NA= Not Applicable)

Supplementary Table 4. Newcastle-Ottowa Scale (NOS) tool for risk of bias detection in non-randomized trials.

Study characteristics examined: (1) representativeness of exposed cohort, (2) selection of nonexposed cohort, (3) exposure assessment, (4) outcome of interest not present at the start of the study, (5)

comparability of cohorts, (6) outcome assessment, (7) adequacy of length of time before follow-up, and (8) adequacy of follow-up of cohorts

Supplementary Figure 1. Funnel plots of JAK-inhibitor treatment for (A) Mortality, (B) ICU Admission, (C) Requirement of Mechanical Ventilation, (D) ARDS, and (E) Discharge

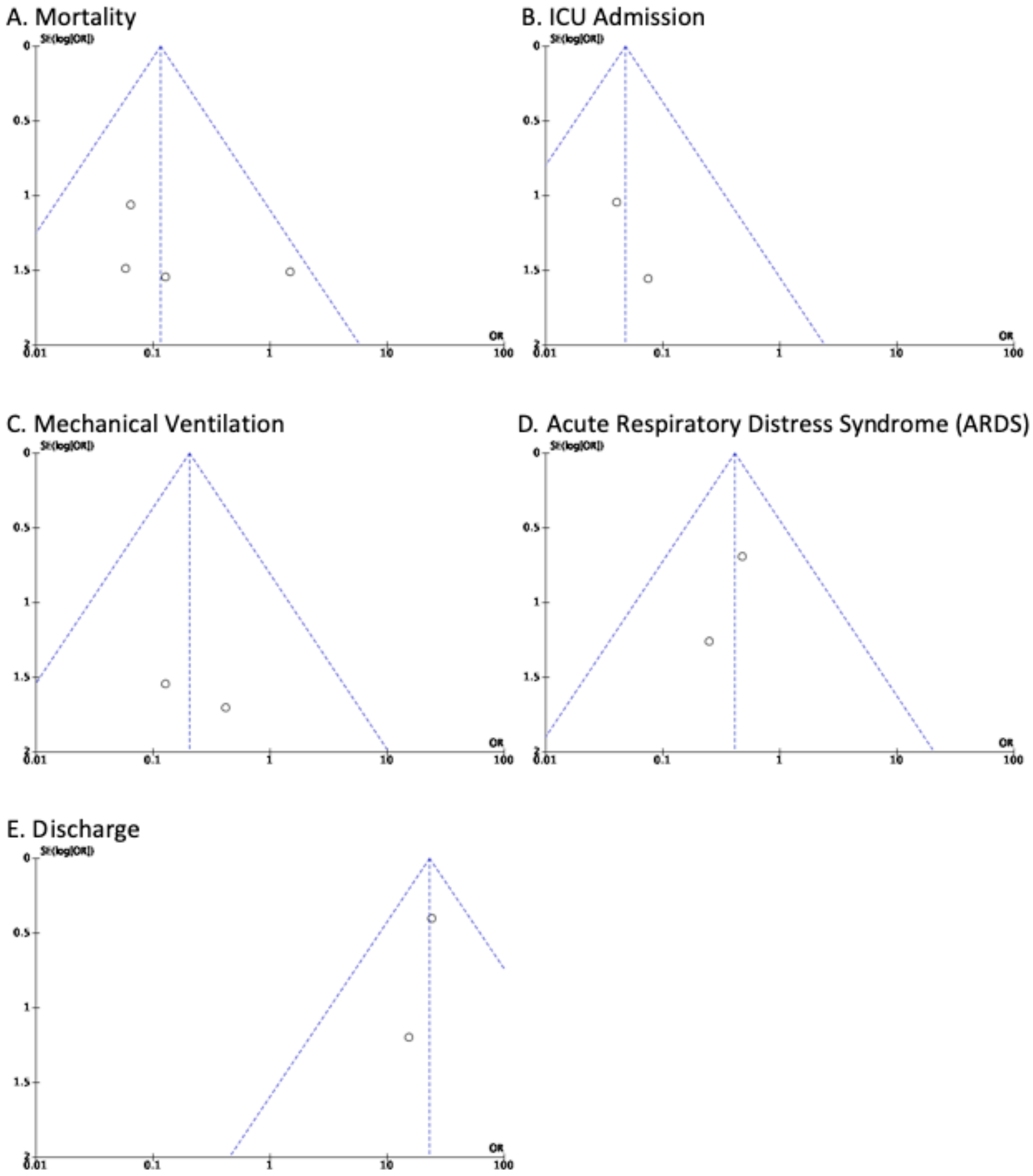

Supplementary Figure 2. Funnel plots of Type I interferon treatment for (A) Mortality, (B) ICU Admission, (C) Requirement of Mechanical Ventilation, (D) Severe or Critical Disease, and (E) Discharge.

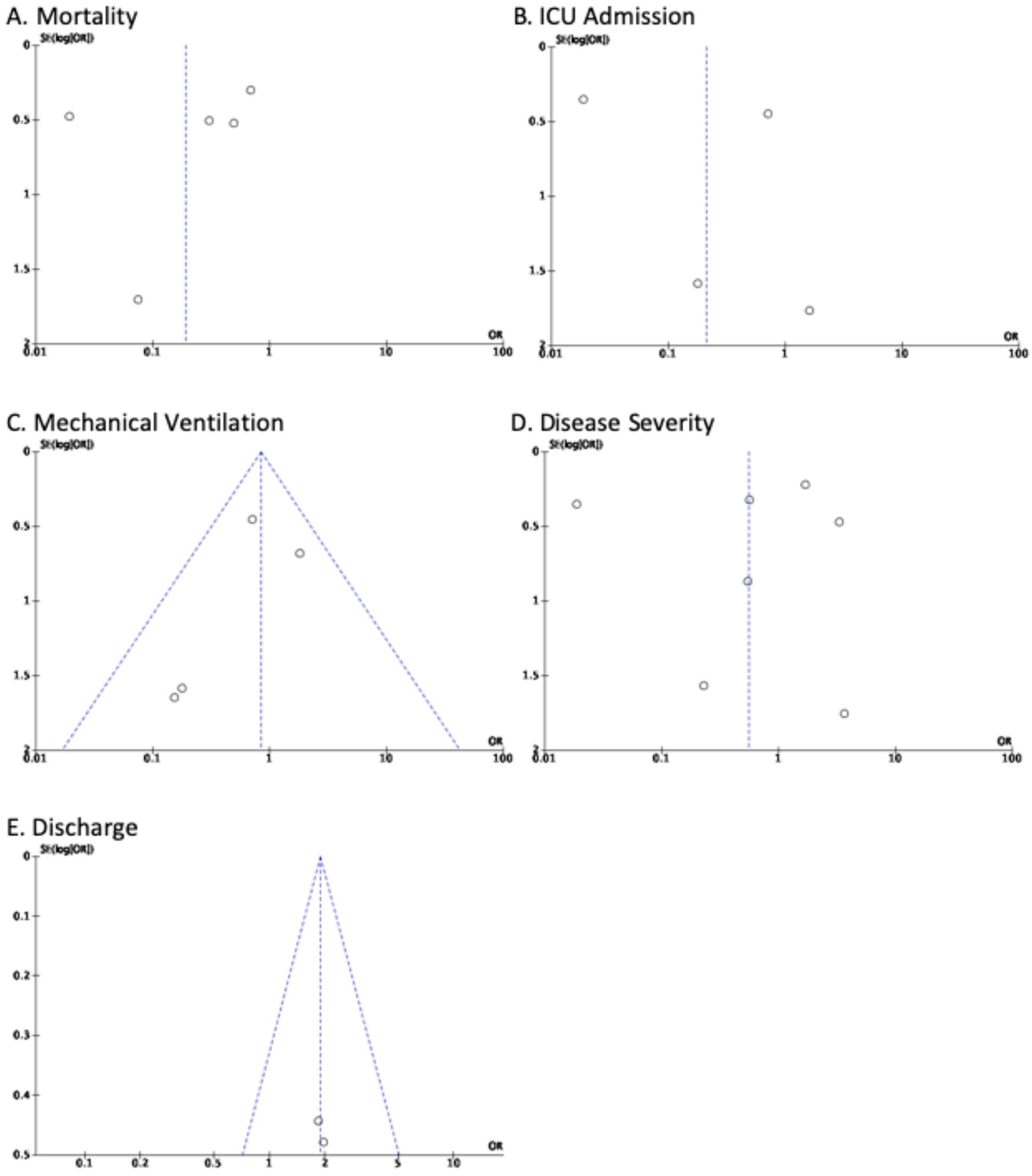

Supplementary Table 1. Total outcome data stratified by included study

First Author, Year	Total Deaths	Cases where IMV was required	Cases admitted to ICU	Total Severe + Critical Cases	ARDS	Discharge
Bronte 2020	26	*	*	*	18	*
Cantini 2020a	5	*	15	*	*	98
Cantini 2020b	0	*	4	*	*	8
Cao 2020	3	3	*	*	*	*
Giudice 2020	2	1	*	*	5	*
Chen 2020	*	*	*	50	*	*
Davoudi-Monfared 2020	25	32	36	*	*	54
Du 2020	1	3	3	4	*	*
Estébanez 2020	63	*	*	*	*	*
Fan 2020	*	*	*	8	*	*
Hung 2020	0	1	*	*	*	*
Liu 2020	*	0	3	5	*	*
Pereda 2020	24	*	82	82	*	151
Wang 2020	16	*	*	111	*	*
Zhou 2020	*	12	*	35	*	*

* Data was not reported across control and treatment group or not clear

Supplementary Table 2. Definition of a severe or critical case in included studies for which that measure was analyzed.

<table>
<tr><th>First Author, Year</th><th>Definition of Severe + Critical Case</th></tr>
<tr><td>Chen 2020</td><td>One of the following: (1) respiratory distress, respiratory rate ≥ 30 times/min; (2) fingertip oxygen saturation ≤ 93% in resting state; (3) arterial oxygen partial pressure/fraction of inspired oxygen ≤300 mmHg.</td></tr>
<tr><td>Davoudi-Monfared 2020</td><td>(1) hypoxemia (need for noninvasive or invasive respiratory support to provide capillary oxygen saturation above 90%) (2) Hypotension (systolic blood pressure less than 90 mmHg or vasopressor requirement) (3) renal failure secondary to COVID-19 (according to KDIGO definition) [18] (4) neurologic disorder secondary to COVID-19 (decrease of 2 or more scores in Glasgow Coma Scale) (5) thrombocytopenia secondary to COVID-19 (platelet count less than 150000 /mm3) (6) severe gastrointestinal symptoms secondary to COVID-19 (vomiting/diarrhea that caused at least mild dehydration).</td></tr>
<tr><td>Du 2020</td><td>One of the following criteria: (1) shortness of breath with increased respiratory rate (RR) except for the influence of fever and crying (RR 60 breaths per minute for those younger than 2 60 months, RR 50 breaths per minute for those aged between 2 and 12 months, RR 40 breaths per minute 61 for those aged between 1 and 5 years, and RR 30 breaths per minute for those older than 5 years); (2) oxygen saturation 92% at rest; (3) hypoxia with accessory respiration (groaning, flaring of nares, three concave sign), cyanosis, and intermittent apnea; (4) disturbance of consciousness with somnolence and convulsions; (5) food refusal or feeding difficulty, with signs of dehydration; (6) high-resolution CT showing bilateral or multi-lobe involvement, with rapid aggressiveness or pleural effusion.</td></tr>
<tr><td>Fan 2020</td><td rowspan="2">One of the following criteria: (1) Respiratory distress (≧30 breaths/ min); (2) Oxygen saturation≤93% at rest; (3) Arterial partial pressure of oxygen (PaO2)/ fraction of inspired oxygen (FiO2)≦ 300mmHg (l mmHg=0.133kPa). In high-altitude areas (at an altitude of over 1,000 meters above the sea level), PaO2/ FiO2 shall be corrected by the following formula: PaO2/ FiO2 x[Atmospheric pressure (mmHg)/760] Cases with chest imaging that shows obvious lesion progression within 24-48 hours >50% shall be managed as severe cases. Child cases meeting any of the following criteria: (1) Tachypnea (RR ≥ 60 breaths/min for infants aged below 2 months; RR ≥ 50 BPM for infants aged 2-12 months; RR ≥ 40 BPM for children aged 1-5 years, and RR ≥ 30 BPM for children above 5 years old) independent of fever and crying; (2) Oxygen saturation ≤ 92% on finger pulse oximeter taken at rest; (3) Labored breathing (moaning, nasal fluttering, and infrasternal, supraclavicular and intercostal retraction), cyanosis, and intermittent apnea; (4) Lethargy and convulsion; (5) Difficulty feeding and signs of dehydration.</td></tr>
<tr><td>Wang 2020</td></tr>
<tr><td>Liu 2020</td><td>One of the following: resting oxygen saturation less than 93% or arterial partial oxygen pressure <60 mm Hg or respiratory rate more than 30 breaths per minute.</td></tr>
<tr><td>Pereda 2020</td><td>Artificial Ventilation, ARDS</td></tr>
<tr><td>Zhou 2020</td><td>One of the following: resting oxygen saturation less than 93% or arterial partial oxygen pressure <60 mm Hg or respiratory rate more than 30 breaths per minute.</td></tr>
</table>

* Information was not presented

Supplementary Table 3. RoB 2 check list for detection of bias in randomized trials.

		Cao 2020	Davoudi-Monfared 220	Giudice 2020	Hung 2020
**Bias due to randomization**					
**1.1**	Was the allocation sequence random?	Y	Y	Y	Y
**1.2**	Was the allocation sequence concealed until participants were enrolled and assigned to interventions?	PY	NI	NI	N
**1.3**	Did baseline difference between intervention groups suggest a problem with the randomization process?	N	PN	NI	N
	**Risk-of-bias judgement**	Low	Some Concerns	Some Concerns	High
	Optional: What is the predicted direction of bias arising from the randomization process?				
**Bias due to deviation from assignment to intervention**					
**2.1**	Were participants aware of their assigned intervention during the trial?	N	NI	NI	Y
**2.2**	Were carers and people delivering the interventions aware of the participants' assigned intervention during the trial?	Y	NI	NI	Y
**2.3**	**If Y/PY/NI to 2.1 or 2.2:** Were there deviations from the intended intervention balanced between groups	N	PN	N	PN
**2.4**	**If Y/PY to 2.3:** Were these deviations likely to have affected the outcome?	NA	NA	NA	NA
**2.5**	**If Y/PY/NI to 2.4:** Were these deviations from intended intervention balanced between groups?	NA	NA	NA	NA
**2.6**	Was an appropriate analysis used to estimate the effect of assignment to intervention?	Y	PY	Y	Y
**2.7**	**If N/PN/NI to 2.6:** Was there potential for a substantial impact (on the result) of the failure to analyze participants in the group to which they were randomized?	NA	NA	NA	NA
	**Risk-of-bias judgement**	Low	Low	Low	Low
	Optional: What is the predicted direction of bias arising from the randomization process?				
**Bias due to deviation from adherence to intervention**					
**2.1**	Were participants aware of their assigned intervention during the trial?	N	NI	NI	Y
**2.2**	Were carers and people delivering the interventions aware of participants' assigned intervention during the trial?	Y	NI	NI	Y

**2.3**	**If Y/PY/NI to 2.1 or 2.2:** Were important non-protocol interventions balanced across intervention groups?	NI	NI	PN	NI
**2.4**	Were there failures in implementing the intervention that could have affected the outcome?	PN	PN	PN	PN
**2.5**	Was there non-adherence to the assigned intervention regimen that could have affected participants' outcomes?	NI	NI	NI	NI
**2.6**	**If N/PN/NI to 2.3, or Y/PY/NI to 2.4 or 2.5:** Was an appropriate analysis used to estimate the effect of adhering to the intervention?	PY	PY	PY	Y
	**Risk-of-bias judgement**	Some Concerns	Some Concerns	Some Concerns	Some Concerns
	Optional: What is the predicted direction of bias arising from the randomization process?				
**Bias due to missing outcome data**					
**3.1**	Were data for this outcome available for all, or nearly all, participants randomized?	Y	Y	N	Y
**3.2**	**If N/PN/NI to 3.1:** Is there evidence that the result was not biased by missing outcome data?	NA	NA	PY	NA
**3.3**	**If N/PN to 3.2:** Could missingness in the outcome depend on its true value?	NA	NA	NA	NA
**3.4**	**If Y/PY/NI to 3.3:** Is it likely that missingness in the outcome depended on its true value?	NA	NA	NA	NA
	**Risk-of-bias judgement**	Low	Low	Low	Low
	Optional: What is the predicted direction of bias arising from the randomization process?				
**Bias due to outcome measurement**					
**4.1**	Was the method of measuring the outcome inappropriate?	N	N	N	N
**4.2**	Could measurement or ascertainment of the outcome have differed between intervention groups?	PN	PN	PN	N
**4.3**	**If N/PN/NI to 4.1 and 4.2:** Were outcome assessors aware of the intervention received by study participants?	Y	NI	NI	Y
**4.4**	**If Y/PY/NI to 4.3:** Could assessment of the outcome have been influenced by knowledge of intervention received?	PY	PY	NI	PN
**4.5**	**If Y/PY/NI to 4.4:** Is it likely that assessment of the outcome was influenced by knowledge of intervention received?	PN	NI	PN	NA
	**Risk-of-bias judgement**	Low	High	Some Concerns	Low
	Optional: What is the predicted direction of bias arising from the randomization process?				

**Bias due to selection of reported result**					
**5.1**	Were the data that produced this result analyzed in accordance with a pre-specified analysis plan that was finalized before unblinded outcome data were available for analysis?	Y	Y	Y	Y
**5.2**	Is the numerical result being assessed likely to have been selected, on the basis of the results, from multiple eligible outcome measurements (e.g. scales, definitions, time points) within the outcome domain?	N	N	N	N
**5.3**	Is the numerical result being assessed likely to have been selected, on the basis of the results, from multiple eligible analyses of the data?	N	N	N	N
	**Risk-of-bias judgement**	Low	Low	Low	Low
	Optional: What is the predicted direction of bias arising from the randomization process?				
**Overall Risk-of-Bias**					
	**Risk-of-bias judgement**	Low	Some Concerns	Low	Low
	Optional: What is the predicted direction of bias arising from the randomization process?				

(N=No; PN=Probably No; Y=Yes; PY= Probably Yes; NI= Not Indicated; NA= Not Applicable)

Supplementary Table 4. Newcastle-Ottowa Scale (NOS) tool for risk of bias detection in non-randomized trials.

Study	Outcome	Selection domain (maximum 4 stars)	Comparability domain (maximum 2 stars)	Outcome domain (maximum 3 stars)
Bronte 2020	Mortality	4	0	2
Bronte 2020	ARDS	3	0	2
Cantini 2020a	Mortality	4	0	2
Cantini 2020a	ICU Admission	4	0	3
Cantini 2020a	Discharge	4	0	3
Cantini 2020b	Mortality	4	0	2
Cantini 2020b	ICU Admission	4	0	3
Cantini 2020b	Discharge	4	0	3
Chen 2020	Disease Severity	3	0	3
Du 2020	Mortality	4	0	3
Du 2020	ICU Admission	3	0	3
Du 2020	Mechanical Ventilation	3	0	3
Estébanez 2020	Mortality	4	0	3
Fan 2020	Disease Severity	3	0	3
Liu 2020	ICU Admission	4	0	2
Liu 2020	Mechanical Ventilation	4	0	2
Liu 2020	Disease Severity	3	0	2
Pereda 2020	Mortality	4	0	1
Pereda 2020	ICU Admission	4	0	1
Pereda 2020	Disease Severity	3	0	1
Pereda 2020	Discharge	4	0	1
Wang 2020	Mortality	4	0	3
Wang 2020	Disease Severity	3	0	3
Zhou 2020	Mechanical Ventilation	4	0	3
Zhou 2020	Disease Severity	4	0	3

ARDS = Acute Respiratory Distress Syndrome
